# York Cottage Hospital

**Published:** 1895-08-31

**Authors:** 


### The Institutional Workshop.

### WITHIN THE HOSPITALS.

### YORK COUNTY HOSPITAL.

(By Our Special Commissioner.)

The York County Hospital has been famous for its surgeons in bygone days, a fact which says more for surgery than may at first appear. The institution has always suffered from defective construction; the results of these defects were as patent when we visited it in the beginning of the present month as on the day when it was opened. As originally constructed, this hospital contained a combined system of warming and ventilation, which was exclusively relied upon for keeping the ward air up to its proper standard of purity. To prevent the possibility of the air of heaven entering this benighted building through the usual channels, the windows were simply glazed panels without means of opening, and there were no open fireplaces in any of the wards. For nine years the complaints of the close and offensive state of the ward air were disregarded, but at last wound diseases prevailed, and increased to such an extent that the surgeons found it impossible to make the smallest incision without erysipelas supervening. Then the medical staff took combined action, which led to the closing of the hospital, the abolition of the artificial system, and the substitution for it of open windows and fireplaces. The whole of the hospital was then thoroughly cleansed, and as a result erysipelas as a hospital disease has disappeared, and operations as a whole do well. We commend this experience and the warning it conveys to the close attention of hospital authorities everywhere in this country, because we are painfully aware that certain people who, it must be remembered, strive to make their living by the introduction of artificial ventilation into British hospitals are striving in season and out of season to induce those responsible for new hospitals in the present day to adopt it in this climate, to which it is certainly unsuited, and where it has proved such a ghastly failure in the past. At the time of our visit the condition of the atmosphere in the children's ward and the accident ward on the ground floor was unsatisfactory. There can be no doubt that steps should be taken to secure cross-ventilation in these wards. We are further of opinion that it is desirable that the windows should be lowered, and that air should be brought in from outside by flues placed under the floors, which should open under each of the beds which are arranged along the blank wall opposite the windows.

#### General Condition of the Wards.

We visited the hospital under somewhat unfavourable conditions, as it was barely out of the hands of the cleaners and decorators, and several of the staff were away. So far as we could judge, evidence exists of the earnest endeavour of the officers and staff to maintain the institution in a state of efficiency. In a few wards teak floors appeared to have been laid down, and it would be well if steps were taken to introduce them throughout the building. We think that more attention should be paid to the furniture and fittings, which have done the hospital good service during the forty odd years of its existence, and which might with advantage be renewed largely, if not altogether. One small matter may be mentioned here that affects the comfort of the residents, and that is the circumstance that the residents' rooms might be made more comfortable and attractive than they are at present. The house surgeon's room, for instance, requires a new carpet, and we hope it may be voted at the next meeting of the House Committee. We are aware that there is a feeling among some of the managers at the present time that they must be strictly economical as the funds at their command are limited, and we have therefore taken care not to make these recommendations before studying the balance-sheet. It is no doubt desirable that the annual subscriptions should increase, although it is satisfactory to find that they have been maintained at a fairly uniform amount down to the year 1894, when there was a falling off of about £40. This is evidently an institution where the managers are bent upon accumulating considerable funds. It appears that the income derived from dividends and rents has increased from about £1,300 in 1858 to £1,850 in 1871, to £2,560 in 1881. They stood at £2,570 in 1892, and fell to £2,408 in 1894. The amount of money invested each year has varied from £1,800 in 1872 to £1,000 in 1893, upwards of £10,000 having been invested in the last ten years. The same spirit of thrift is shown in connection with the nursing department, the nursing fund investments account showing a capital sum of £3,200. We should be the last persons in the world to discourage thrift and the careful husbanding of resources, but there is a limit, which, if passed, causes thrift to degenerate into parsimony, to the injury of an institution. We are of opinion that a consideration of this view might well occupy the immediate attention of the managers of the York County Hospital, because it should lead to the selling out of a certain amount of stock in order to provide adequate fittings and accommodation to secure a maximum of efficiency throughout the hospital.

#### Accommodation for the Nurses and the General Fittings.

We have already suggested that it is desirable that the whole of the fittings throughout the hospital should be carefully gone over with a view to their renewal. No very large sum would be involved in such necessary work, and a wise expenditure in this direction must tend materially to improve the comforts of the patients, the efficiency of the staff, and the hygiene of the wards and offices. The least satisfactory portion of the present buildings is that set apart for the accommodation of the nursing staff. Here are to be seen some of the smallest and worst constructed cubicles we have ever met with in the course of our inspections. The arrangements in this branch of the hospital are unworthy of a great county institution, and, seeing that considerable sums of money are taken each year from probationers who go to the county hospital to be trained, no time should be lost in condemning the present arrangements, and taking steps to erect a nursing home upon the newest and best principles.

Until this is done, nurses will be wise to avoid the York County Hospital, because it is far and away behind the majority of institutions which lay themselves out to train nurses, and take fees from probationers for their instruction. We have no doubt that what is here said has already commended itself to the more intelligent of the managers, and that they will welcome this frank and friendly criticism because it should assist them to set on foot the necessary building operations which have been too long delayed. When the York County Hospital has been so renovated and reconstructed, when it has been provided with an adequate nursing home, and when the ground floor wards, their ventilation and hygienic condition have been modernised and materially altered, then, and not till then, will this institution be worthy to continue the useful work it has done for the people of York and the neighbourhood since the year 1849. We shall be very glad to give the committee any help or assistance in our power with a view to speedily remedying the defects we have felt it our duty to point out. It is a pleasure to add that there is an evident desire on the part of the staff to do everything in their power to promote the speedy recovery and well-being of the patients. We were further glad to see that the hospital has provided an ambulance for accident cases, which they have placed at the disposal of the local police, and so conferred a considerable boon upon the inhabitants of the City of York. We understand, however, that they fail to appreciate this advantage at present from want of knowledge, which the local press will, no doubt, promptly supply.

